# Intelligence and persisting with medication for two years: Analysis in a randomised controlled trial

**DOI:** 10.1016/j.intell.2009.01.001

**Published:** 2009-11

**Authors:** Ian J. Deary, Catharine R. Gale, Marlene C.W. Stewart, F. Gerald R. Fowkes, Gordon D. Murray, G. David Batty, Jacqueline F. Price

**Affiliations:** a Department of Psychology, School of Philosophy, Psychology and Language Sciences, University of Edinburgh, 7 George Square, Edinburgh EH8 9JZ, UK; b Medical Research Council Centre for Cognitive Ageing and Cognitive Epidemiology, UK; c MRC Epidemiology Resource Centre, University of Southampton, UK; d Division of Community Health Sciences, School of Clinical Sciences and Community Health, University of Edinburgh, UK; e MRC Social and Public Health Sciences Unit, University of Glasgow, UK

**Keywords:** Intelligence, IQ, Health behaviours, Compliance, Randomised controlled trial, Aspirin, Cognitive epidemiology

## Abstract

The study examined whether verbal intelligence is associated with persisting to take medication for up to two years. The design is a prospective follow-up of compliance with taking medication in high-risk individuals participating in a randomised, placebo-controlled trial set in Central Scotland. Participants were 1993 people aged between 50 and 77 years with an ankle brachial index ≤ 0.95. The medication was 100 mg aspirin or placebo daily.

The principal outcome measure was continuing with taking medication or stopping it due to having ‘changed one's mind’. Higher verbal intelligence was associated with a greater likelihood of continuing to take medication up to two years after randomisation. For a standard deviation increase in Mill Hill Vocabulary Scale score, risk of stopping medication in the first two years of the study was 0.75 (95% CI 0.64 to 0.87, *p* < 0.001). Comparing the highest and lowest quartiles of IQ, the lowest IQ group's relative rate of stopping medication was 2.51 (95% CI 1.52 to 4.22). The effect was not attenuated after adjustment for sex, smoking, or level of deprivation. Verbal intelligence is associated with continuing, medium-to-long term engagement with health self-care, even in the face of uncertainty about whether active treatment is being received, whether the treatment is known to be effective in general, and whether it will be helpful to the individual taking it. Such persisting with potentially helpful health behaviours in the face of uncertainty might partly explain why people with higher intelligence live longer and suffer less morbidity from chronic diseases.

## 1 Introduction

Higher intelligence (IQ), measured in childhood or early adulthood, is associated with living longer (Batty, Deary, & Gottfredson, 2007). The emerging field of cognitive epidemiology aims to establish the limits of this association, uncover its mechanisms, and explore its implications (Deary & Batty, 2007). One of the favoured explanations is that intelligence influences chronic disease onset and ultimately survival via its association with health behaviours (Gottfredson, 2004). This view conceptualises health self-care as a complex set of tasks that require knowledge, decision-making, planning, and engagement. It is hypothesised that people with higher intelligence will manage health self-care more effectively. Supporting evidence from self-reports shows that people with higher childhood intelligence tend to exercise more and have diets that accord better with health information, are less likely to smoke or to be obese or overweight, and have fewer hangovers from drinking alcohol (Batty, Deary, Schoon, & Gale, 2007a,b; Batty, Deary, & Macintyre, 2006, 2007).

These findings suggest that people with higher intelligence tend to behave more healthily, perhaps being better able to take a long view about the implications of their behaviour. They are largely derived from community-based samples, most of which were too young to be at high risk of age-related chronic disease. In the present study we shall inquire whether intelligence influences self-care and disease management in individuals with a chronic pathology who are at moderately increased risk of dying at a younger age. The setting is a community-based, double-blind, randomised clinical trial of subjects screened for generalised atherosclerosis using the ankle brachial index (ABI) (Price et al., 2008). Individuals with an increased risk of cardiovascular events and all-cause mortality—as indicated by a ratio of systolic blood pressure in the ankle to that in the arm (ABI) below a pre-determined cut-off (Ankle Brachial Index Collaboration, 2008)—were randomised to daily low-dose aspirin or placebo. The present study investigates whether higher intelligence, measured shortly after randomisation into the trial, predicted longer-term compliance with study medication for up to two years in individuals who knew themselves to be at relatively high risk of cardiovascular disease.

There is already some information on the factors that affect older people's compliance with medication, especially in randomised controlled trials. Some of this research has concentrated on how compliance varies as a result of the design of trials and the different interventions used to enhance compliance (Russell, Conn, & Jantarakupt, 2006). However, there is variation in compliance even within single trials, where all participants are exposed to the same regimen of treatment and encouragements to comply. This motivated the research for person-level predictors of compliance. A large quantitative review of compliance with medical recommendations found that older age, female gender, higher income (but not general socio-economic status), and education were associated with better compliance (DiMatteo, 2004). The review lacked information on intelligence. It cannot be assumed that intelligence—including verbal intelligence, which will be tested here—is acting as a proxy for education. Although they are strongly correlated, verbal intelligence is highly heritable, and large-scale longitudinal data show that verbal intelligence at age 11 strongly predicts educational outcomes at age 16 (Deary, Spinath, & Bates, 2006; Deary, Strand, Smith, & Fernandes, 2007).

It has been suggested that both verbal intelligence and education are partly-independent contributors to better health literacy (Paasche-Orlow & Wolf, 2007). Health literacy is, in turn, posited to be a mediator of the influence of intelligence and education on good compliance (Paasche-Orlow & Wolf, 2007). However, findings concerning health literacy and compliance are not univocal, and it is possible that some so-called health literacy measures are likely to be acting as little more than verbal ability measures. In a study of very brief (3-day) compliance with anti-retroviral therapy, HIV-infected patients with low health literacy were more likely to show adherence (Paasche-Orlow et al., 2006). The measure of health literacy was the REALM, which tests people on their ability to pronounce 66 medically-related words. Of course, this measure is likely to be related highly to general vocabulary, which will be tested in the present study as the independent variable. In a separate study, both education and health literacy were significant, independent predictors of very brief adherence to combination antiretroviral therapies in HIV patients (Kalichman, Ramachandran, & Catz, 1999). In this case, the health literacy measure was the Test of Health Literacy in Adults, which involves understanding written passages and numerical information concerning health care situations. These studies provide examples of two important omissions in our knowledge concerning the adherence aspect of personal health management. First, intelligence is arguably a missing, more fundamental variable, which is prior to, and a strong determinant of, education and health literacy. Therefore, associations between intelligence and adherence to medications should be examined. Second, the adherence in the above studies is assessed over very short time spans: good health management involves longer-term adherence to behaviours that are beneficial to health. This is especially true among older people where common chronic illnesses, such as cardiovascular disease, account for a substantial proportion of the morbidity and mortality burden.

The present study will examine the association between measured intelligence and a particular aspect of compliance with health care within a randomised clinical trial. The participants' intelligence was assessed on average about three months into the trial. By the time participants had reached the stage where they took the mental test (the Mill Hill Vocabulary Scale), they had already made a number of health-related decisions in the affirmative. First, they responded positively to the initial contact by the study team. Second, they attended a non-GP, non-hospital-based medical research clinic to be assessed. Third, they agreed to be, and were, randomised to aspirin or placebo treatment. Fourth, they had persisted with medication for about three months. Fifth, they agreed to take the Mill Hill Vocabulary Scale. Therefore, in terms of a model of health-based decision making, they had already proceeded a long way. Much of health behaviour and behaviour change research would focus on the factors that made people make some of these choices—and intelligence might be associated with any or all of them—but health self-care with respect to chronic disease involves a long-standing commitment to experts' recommendations regarding optimal health behaviours (Gottfredson, 2004). In the men and women we shall study here, we focus on their persistence with treatment over the next 21 months of the study—after a number of ‘good’ initial decisions—that was associated with intelligence.

Further particularities of the study setting make the study valuable and novel within cognitive epidemiology. Participants within the trial are deciding whether or not to continue a medication for a theoretical benefit. Their blood pressure readings suggest a possible future problem, but not one that is as yet symptomatic, and the risks are communicated to them. They are not certain to be taking the active medication; they have a 50% chance of being on placebo. In this regard, taking part in the trial might be driven by altruism to some extent, because persisting with the medication could add to the greater good by adding to medical knowledge, and verbal intelligence might be related to that aspect. They are aware that it is not known whether even the active medication is effective in reducing risk of cardiovascular disease in people at risk as defined by their ABI, which is why the study was being conducted. It is not certain that, even if they were taking the active medication and it was effective overall in trials, it would be helpful to them individually. The medication could have side effects. In the face of all this uncertainty, they were expected to take the medication daily.

In summary, this study explores whether intelligence is among the factors that influence long-term compliance in a situation where the risk of illness and the likelihood of treatment being present and effective are uncertain. This is likely to be representative of many pro-health decisions, which must be taken in the face of uncertain evidence and then persisted with. Because of the information gathering and decision making required, and the subsequent requirement to aim at a distant goal, we hypothesise that intelligence differences will be important.

## 2 Methods

### 2.1 Participants

The Aspirin for Asymptomatic Atherosclerosis (AAA) trial is an on-going double-blind, placebo-controlled, randomised clinical trial that has been conducted in central Scotland (Lanarkshire, Edinburgh and Glasgow), United Kingdom, since 1998 (Price et al., 2008). The International Standard Randomised Controlled Trial Number is ISRCTN66587262. Written informed consent was obtained from all participants prior to the start of the trial. Volunteers aged 50 to 76 years (some were 77 by the time they were tested) were recruited by direct mailing of people registered with participating general practices in the study area. 83% of practices contacted agreed to participate. People were not eligible to attend for screening of their ABI and subsequent randomisation into the trial if they had a previous history of myocardial infarction or stroke, or if they were already taking aspirin or warfarin for any reason. In the study's recruitment information sheet, and at the clinics, subjects were told that, if they had a low ABI, then they were, “one of the people more likely to suffer from heart disease or strokes in the future”.

### 2.2 Assessment of ankle brachial index

Potential participants attended a screening clinic at which their ABI was calculated as the ratio of the lowest systolic blood pressure in either ankle (dorsalis pedis or posterior tibial) to the higher of the left or right arm systolic pressures. Exclusion criteria included: an ABI > 0.95 in both legs; taking aspirin, other antiplatelet or anticoagulant treatment; severe indigestion; clinical cardiovascular disease; chronic liver or kidney disease; undergoing chemotherapy; contraindications to treatment with aspirin; or an abnormally high or low plasma haematocrit on a blood sample taken at the clinic. Eligible participants were assessed for smoking status: current, previous, or never. The deprivation of their area of current residence was assessed using the Carstairs and Morris index (Carstairs & Morris, 1991).

### 2.3 Assessment of verbal intelligence

A baseline measure of cognitive function was made at around 3 months after participants had been randomised to, and started the trial medication (aspirin or placebo). Cognitive function was a secondary outcome of the trial. Participants were asked to complete a version of the Mill Hill Vocabulary Scale, which assesses verbal intelligence (Raven, Raven, & Court, 1998). The version used was the combined 44 synonyms of the Junior and Senior Form A tests. For each item the participants chose which of six answer options was closest in meaning to the target word. The total number of items correct out of a possible 44 was used to indicate verbal intelligence. Verbal intelligence, especially as assessed by vocabulary tests, peaks in mid-life and deteriorates little with age and, therefore, is an indication of a person's level of peak prior ability (Salthouse, 2004; Schaie, 2005).

### 2.4 Assessment of compliance with trial medication

Participants were randomised to aspirin (enteric coated 100 mg daily) or placebo. Both preparations were provided by Bayer HealthCare plc. Participants were reviewed annually either in a research clinic or by telephone. They were also encouraged to contact the trial office at any time if they went into hospital or stopped their study medication. If participants were prescribed aspirin or another antiplatelet drug (including prescription following a cardiovascular event) or started to self-medicate with aspirin, then their study medication was discontinued. If a participant stopped their study medication for any period other than for medical reasons, they were encouraged to re-start. Participants were provided with a diary in which to record whether or not they took their study medication as requested. Self-reported compliance was assessed at each subject's annual review. A specially trained nurse asked questions to determine: whether participants were currently taking their medication; whether they had taken their medication for two thirds or more of the preceding year; and, if they had stopped their medication, on what date this had occurred. Supplies of tablets were renewed annually by post.

If participants had stopped taking medication then a reason for non-compliance and the date of stopping were noted. Reasons for stopping were as follows: ‘changed their mind’, moved away, allergic to aspirin, other aspirin-related symptoms (nosebleed, dyspepsia or indigestion, bruising, blood loss from stomach or gut, ulcer, anaemia), other symptoms, started on aspirin by their General Practitioner, started self-medication with aspirin, started medication contraindicated with aspirin, or death. We did not expect differences between those who persisted with medication and those who stopped for medical reasons. The hypothesis of the present study was based upon the comparison between those who were still complying versus those still alive but who had stopped their study medication because they had ‘changed their mind’; that is, those for whom no medical reason for discontinuing medication could be discerned.

### 2.5 Statistical analyses

Cox proportional hazards regression was used to quantify the associations between the predictor variables and the outcome, which was whether or not participants were still taking medication at 2 years after entering the study. Verbal intelligence, sex, smoking status, and deprivation index were the independent (predictor) variables. Age was not related to stopping medication (HR 1.00, 95% CI 0.97 to 1.02, *p* = 0.78) and had no effect on other estimates of risk when it was included in the models. It was omitted from the models reported here. Time to stopping medication was measured from the day participants took the Mill Hill Vocabulary test. When analyses were repeated using time measured from the point of randomisation the hazard ratio and 95% CI for stopping medication according to Mill Hill Vocabulary Scale score were identical.

## 3 Results

**Fig. 1:**
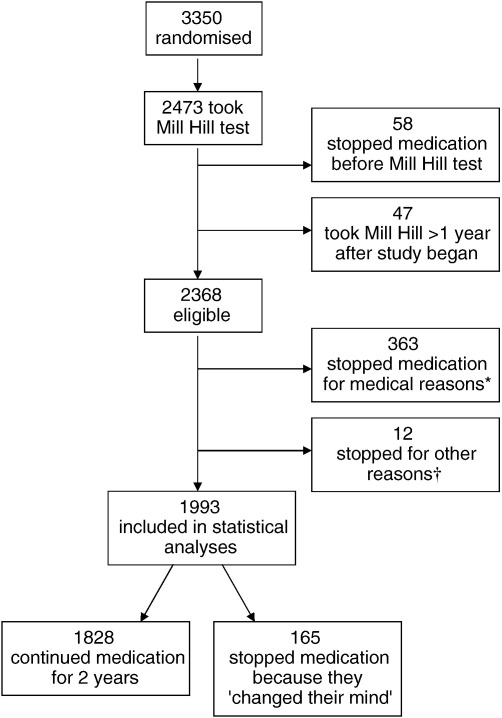
Consort flow chart showing how the analytical sample was derived from the participants who were randomised in the AAA trial. ⁎Aspirin-related symptoms, *N* = 125; other symptoms, *N* = 113; started prescribed aspirin, *N* = 107; self-medicated with aspirin, *N* = 11; started contraindicated medication, *N* = 7. †Moved away, *N* = 4; died, *N* = 8.

Fig. 1 shows how the present study's analytical sample was derived from the 3350 participants who were randomised in the AAA trial. In total, 2368 participants were taking the study medication and agreed to take the Mill Hill Vocabulary Scale, which was administered a mean of 103 (SD = 26.2) days after randomisation. Two years after starting medication there was an analytic sample of 1993 individuals: 1828 were still taking medication and 165 had stopped because they had ‘changed their mind’. In addition, 363 participants had stopped taking the medication for medical reasons, 4 because they had moved away from the area, and 8 subjects had died (see Fig. 1). There was no significant difference in mean Mill Hill Vocabulary Scale score between those who continued to take medication for two years (31.5, SD 4.6), those who given up medication for medical reasons (31.4, SD 4.5), and those who were not taking medication because they had moved away or died (32.0, SD, 3.8). Participants who had given up medication because they had ‘changed their mind’ had a mean Mill Hill Vocabulary score that was significantly lower than those of participants in these other groups (30.1, SD 4.0; *p* < 0.001, Cohen's *d* = 0.32). The analysis that follows is based on the 1993 participants in the two groups who either continued to take medication for two years or who stopped it because they had ‘changed their mind’.

Based on one standard deviation advantage in Mill Hill Vocabulary Scale scores, the hazard ratio (HR) (95% CI, *p*) for stopping medication by two years into the study was 0.75 (0.64 to 0.87, *p* < 0.001). When sex was included in the model the HR for Mill Hill Vocabulary was unchanged, and the effect of sex was non-significant: 1.00 (0.71 to 1.41, *p* = 0.99). In a model which included smoking status, the HR for Mill Hill Vocabulary Scale score was unchanged. In this model smoking was also a significant predictor of stopping medication. Compared with the never-smoked group, the HRs for current smokers and ex-smokers were 1.97 (1.34 to 2.91, *p* < 0.001), and 1.52 (1.02 to 2.25, *p* = 0.04), respectively. In a model which included the deprivation category of the person's current residence, the HR for Mill Hill Vocabulary Scale score was almost unattenuated (0.74, 0.63 to 0.87, *p* < 0.001). In this model deprivation category was not a significant predictor of stopping medication. None of the six more affluent categories differing significantly from the most deprived (category 7) as regards risk of stopping medication (*p* = 0.65).

**Fig. 2:**
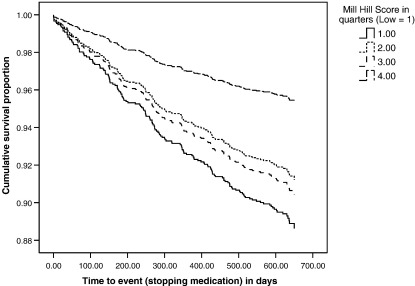
Survival curves for stopping medication according to quarters of the distribution of Mill Hill Vocabulary Scale score.

Fig. 2 shows the survival curves of continuing medication by quartile of Mill Hill Vocabulary Scale score. There were marked differences in the proportion continuing medication between people in the highest quarter of the distribution and those in the remaining three quarters. People in the top and bottom quarters of the distribution had the lowest and highest risks, respectively, of stopping medication during the two-year follow-up. The two middle quartiles of IQ scores fell between these, but not in the order that would indicate a straightforward dose–response effect across the four quartiles. There was a significant trend across the quartiles (*p* = 0.005). By comparison with the highest (fourth) IQ quartile, the relative rates (HR [95% CI, *p*]) of the other three quartiles were: lowest = 2.53 (1.52 to 4.22, *p* < 0.001); second = 1.93 (1.14 to 3.27, *p* = 0.015); third = 2.11 (1.15 to 3.58, *p* = 0.005).

## 4 Discussion

In a randomised, placebo-controlled trial of aspirin for asymptomatic atherosclerosis, people with higher verbal intelligence were less likely to change their mind about continuing to take medication during an observational period of two years. People in the highest quartile of verbal intelligence were especially likely to continue with their medication; by comparison, the people in the lowest IQ quartile had 2.5 times the risk of stopping medication. Sex, smoking and level of deprivation did not attenuate the effects. Being a current or ex-smoker was associated with a significantly increased risk of stopping medication, independently of verbal intelligence. Therefore, whatever else it is related to in health decision-making, intelligence differences are associated with persevering in the medium-to-long term with a course of health actions to which one has already subscribed.

Strengths of the study include the large number of subjects, the double-blind, randomised and placebo-controlled design, and the long follow-up period. It is a feature, and possibly a limitation, of the study that the Mill Hill Vocabulary Scale was not administered until about three months after the randomisation to medication or placebo took place. However, we think this could be a strength, insofar as the decision to take the test was the last in a long line of health-relevant decisions made in the affirmative. The study shows that, even after all of these pro-health decisions, people who just change their minds thereafter have lower average intelligence.

The present study is valuable because it models much of what is important in avoiding the now-common chronic conditions that especially affect older people. There are some health settings where there is an unequivocal and proximal correct course—such as maintaining good glycaemic balance in diabetes in children—and there is evidence that parental intelligence is related to that (Ross, Frier, Kelnar, & Deary, 2001). The present study models a different, more protean part of real life. Health-related decisions have to be made and stood by in the face of uncertainty about whether they are correct, or of benefit to the individual making them. It might be that what characterises intelligence in health self-care is the willingness to persist in playing the odds for a best-guess theoretical benefit, and the taking of a long view as regards behaviour. This style of behaviour might be a consequence of a mental capacity that can understand that there are multiple small risks to health, and appreciate the need to plan one's life to overcome many of them. This type of behaviour is, perhaps, more easily evinced in a social setting that is correlated with higher intelligence. However, if such social mediation was the explanation for our findings then we would expect the strength of the association between intelligence and compliance with medication to be more strongly attenuated by level of deprivation.

The present study can be used as a foundation for future studies. These might add education and measures of health literacy as possible mediators of the influence of intelligence on adherence to treatments in the longer term. However, care would have to be taken with regard to which measures of health literacy are used. These vary considerably in content, which can involve the pronunciation of words, the understanding of written material, or the computation of solutions from numerical data.

It is also important to develop the present study's findings and provide more insight into other person-level variables, including social cognition, that might explain people's health-related decision making. Of particular relevance would be an examination of the role of illness cognitions. Thus, future research could include consideration of the findings that patients' beliefs about medicines are associated with adherence to treatment (Horne & Weinman, 1999). The beliefs that correlate with adherence to treatment include the necessity of taking medicines for the given condition, and the person's concerns about factors such as side effects and dependence. In Horne & Weinman's study these medication beliefs were a stronger predictor of adherence than sociodemographic factors. Intelligence might be a contributing factor to these assessments, and help to explain how patients' analysis of cost/benefit is important in determining compliance with treatment. In the present study we were not able to explore how patients in the trial perceived their risk of possible illness and how the treatment might affect it. It would also be useful in future to investigate how effective patients perceived aspirin to be, and their views of the importance for future health of taking it consistently. Future research might also include a consideration of the role of health locus of control in health-related decision making. For example, women who reported omitting taking medication for breast cancer had lower internal locus of control (Atkins & Fallowfield, 2006). The possible role of locus of control is boosted by the finding that a higher locus of control in childhood contributes to better health outcomes and behaviours at age 30 (Gale, Batty, & Deary, 2008). Moreover, in this latter study the contribution of locus of control was additional to that contributed by higher childhood IQ, and might partly have mediated the effect of childhood IQ.

In conclusion, the present study finds a novel and important aspect of health self-management that is associated with verbal intelligence. Further research should investigate the mechanisms of the association, which will point to ways of promoting optimal health behaviours, irrespective of intelligence level. In addition, the findings might provide a part of the as-yet elusive explanations for the association between intelligence and survival (Whalley & Deary, 2001).
